# Measurement of bone marrow lesions by MR imaging in knee osteoarthritis using quantitative segmentation methods – a reliability and sensitivity to change analysis

**DOI:** 10.1186/1471-2474-15-447

**Published:** 2014-12-20

**Authors:** Flemming K Nielsen, Niels Egund, David Peters, Anne Grethe Jurik

**Affiliations:** Department of Radiology, Aarhus University Hospital, Noerrebrogade 44, 8000 Aarhus, Denmark; Department of Biomedical Engineering, Aarhus University Hospital, Noerrebrogade 44, 8000 Aarhus, Denmark

**Keywords:** Magnetic resonance imaging, Knee osteoarthritis, Bone marrow lesion, DMOADs

## Abstract

**Background:**

Longitudinal assessment of bone marrow lesions (BMLs) in knee osteoarthritis (KOA) by MRI is usually performed using semi-quantitative grading methods. Quantitative segmentation methods may be more sensitive to detect change over time. The purpose of this study was to evaluate and compare the validity and sensitivity to detect changes of two quantitative MR segmentation methods for measuring BMLs in KOA, one computer assisted (CAS) and one manual (MS) method.

**Methods:**

Twenty-two patients with KOA confined to the medial femoro-tibial compartment obtained MRI at baseline and follow-up (median 334 days in between). STIR, T1 and fat saturated T1 post-contrast sequences were obtained using a 1.5 T system. The 44 sagittal STIR sequences were assessed independently by two readers for quantification of BML. The signal intensities (SIs) of the normal bone marrow in the lateral femoral condyles and tibial plateaus were used as threshold values. The volume of bone marrow with SIs exceeding the threshold values (BML) was measured in the medial femoral condyle and tibial plateau and related to the total volume of the condyles/plateaus.

The 95% limits of agreement at baseline were used to determine the sensitivity to change.

**Results:**

The mean threshold values of CAS and MS were almost identical but the absolute and relative BML volumes differed being 1319 mm^3^/10% and 1828 mm^3^/15% in the femur and 941 mm^3^/7% and 2097 mm^3^/18% in the tibia using CAS and MS, respectively. The BML volumes obtained by CAS and MS were significantly correlated but the tissue changes measured were different. The volume of voxels exceeding the threshold values was measured by CAS whereas MS included intervening voxels with normal SI.

The 95% limits of agreement were narrower by CAS than by MS; a significant change of relative BML by CAS was outside the limits of -2.0%-4.7% whereas the limits by MS were -6.9%-8.2%. The BML changed significantly in 13 knees using CAS and in 10 knees by MS.

**Conclusion:**

CAS was a reliable method for measuring BML and more sensitive to detect changes over time than MS. The BML volumes measured by the two methods differed but were significantly correlated.

**Electronic supplementary material:**

The online version of this article (doi:10.1186/1471-2474-15-447) contains supplementary material, which is available to authorized users.

## Background

Osteoarthritis (OA) is the most prevalent form of arthritis [1] and the knee is the most common site of OA in larger synovial joints [2]. Both OA and other arthritides are pathoanatomically characterised by multi-tissue involvement [3]. Clinically, knee osteoarthritis (KOA) can be distinguished from other forms of arthritides using the ACR (American College of Rheumatology) criteria [4] and the radiographic features are different. Early focal joint space narrowing or focal cartilaginous lesions and osteophytes in OA allow differentiation from the generalised cartilage destruction and erosions in rheumatoid arthritis [5, 6]. Magnetic resonance imaging (MRI) of OA and other arthritides share many pathological traits, the most prominent abnormalities being synovitis, joint effusion and bone marrow lesions (BMLs) [7, 8]. The association between these abnormalities and clinical OA features is still uncertain. A meta-analysis by Yusuf et al. [8] systematically reviewed existing literature until March 2010 regarding associations between a variety of MRI findings and pain. Only BML and synovitis/joint effusion were associated with pain, but the level of evidence was moderate [8]. However, BML has been associated with cartilage loss and/or subchondral bone attrition in several studies [9–14].

BMLs are described as ill-defined areas of high signal intensity (SI) on T2-weighted, fat saturated or short tau inversion recovery (STIR) MR images [3]. BMLs are similarly visible by contrast enhanced MRI [11, 15] and histologically they represent a number of non-characteristic abnormalities including fibrovascular tissue [16, 17]. The most widely used scoring methods for severity of BMLs in KOA are confined to semi-quantitative methods [3, 18–20]. In these scoring systems the volume or size of BMLs is graded in steps of 0 – 3 relative to the volume or area of subregions of the subchondral bone marrow. All of these scoring systems have been reported to have excellent reader reliability following local reader training and calibration. However, assessment of the size of lesions with ill-defined margins is subjective and may vary between studies and centers. These considerations may also concern volumetric measurements of BMLs [14, 21–23].

Quantitative MRI measurements with segmentation of BMLs [15, 24] have attracted little interest until recently when three different methods were introduced [25–27]. Compared to semi-quantitative scoring methods, these methods seemingly have a higher reliability and may thus be more sensitive to detect changes over time; so far, this has only been investigated in a few studies. Felson et al. focused on patello-femoral OA [27] and used manual segmentation to demonstrate significant BML changes within periods of 6-12 weeks. To our knowledge, similar quantitative analyses of sensitivity to detect changes have not been performed on the femoro-tibial joint although it could be valuable in emerging studies of disease modifying osteoarthritis drugs (DMOAD) [28, 29].

The purpose of this study was to evaluate and compare the validity and sensitivity to detect changes of two quantitative MR segmentation methods for measuring BMLs in KOA, one computer assisted and one manual method.

## Methods

### Patients

Data was retrieved from a previous randomized placebo-controlled trial of 337 patients with KOA comparing five intra-articular injections of Hyalgan® and placebo, respectively [30]. Ninety patients obtained MRI of the knee and 83 participated in both baseline and follow-up MRI. They all gave written informed consent prior to participating in the study, including consent for publication of their individual details and accompanying images [31]. The study was approved by the Central Region Denmark Committee on Biomedical and Research Ethics. The results of the MR examinations have not previously been published except in a thesis where pooled data were correlated to the clinical findings [31]. The baseline and follow-up MR examinations from 22 patients with medial KOA according to the ACR criteria [32] were randomly selected among the 83 patients [31]. There were 18 females and 4 males; mean age 61 years (41 – 79 years). The median time interval between baseline and follow-up MRI was 334 days (91 – 375 days). Weight bearing radiographs were obtained in all patients and the radiographic grade of medial femoro-tibial OA at baseline varied between 0 and 3 (median/mean 2/2) using Ahlbäck grading [33] with only two knees showing grade 0 and five knees grade 3 changes.

### Imaging

**Figure 1:**
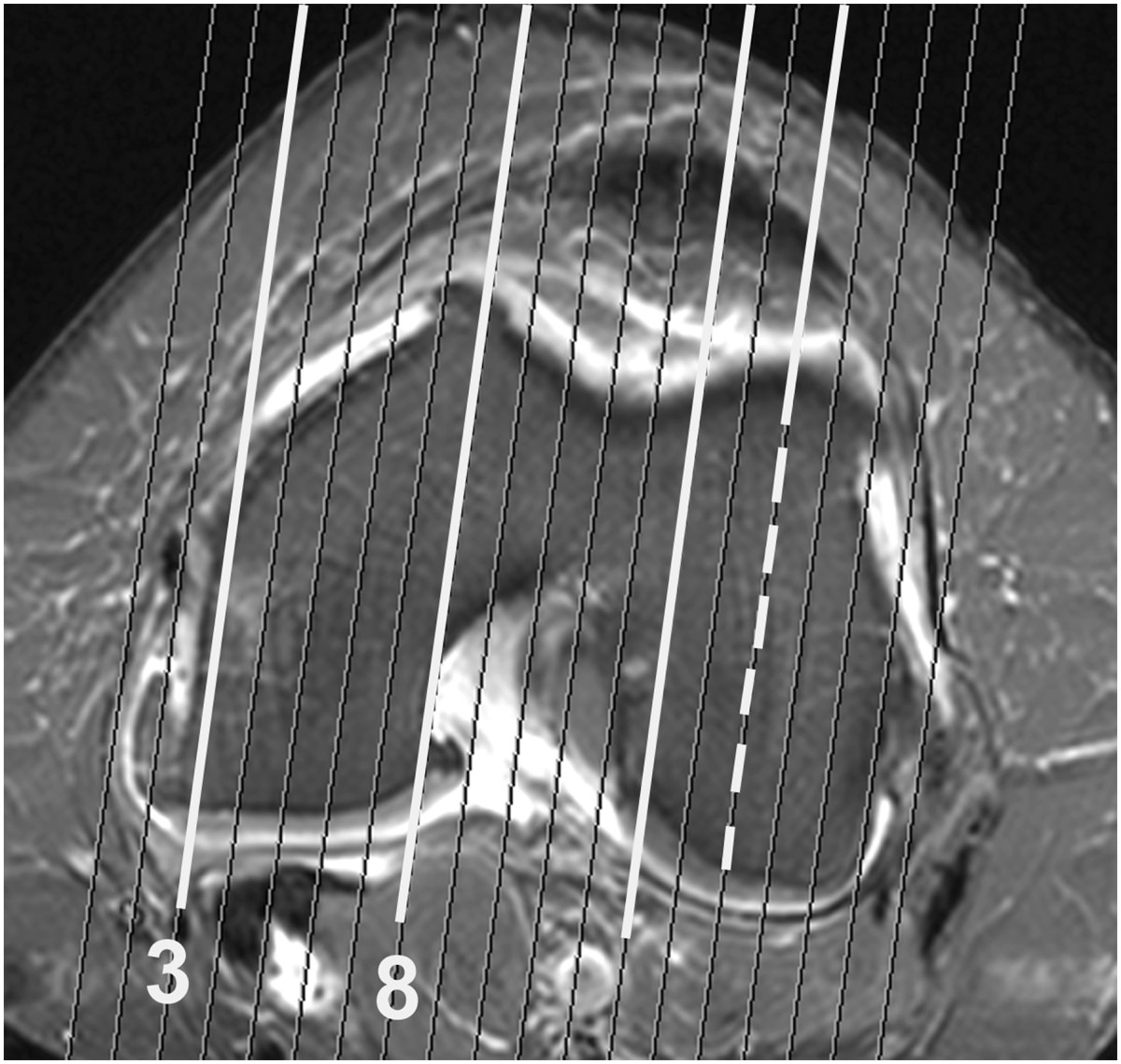
**Trans-axial post-contrast T1FS MR image of the femoral condyles showing the direction of sectioning perpendicular to a line joining the dorsal aspects of the medial and lateral femoral condyle with a width of 4.4 mm.** Optimally, the central slice of the lateral femoral condyle (broken line) is used for estimating the reference values, See Figure 2. Sections adjacent to the inter-condylar area and at the joint margins may contain partial volume information from soft tissue structures (white lines). Four sections of the medial femoral condyle (between section 3 and 8) are available for the measurements of bone marrow lesions (BMLs). Note that the medial condyle decreases ventrally resulting in decreasing bone marrow area on sagittal images medially.

MR examinations were performed using a 1.5 Tesla system (Vision, Siemens, Erlangen, Germany) and a transmit receive four-channel knee coil. The baseline and follow-up examinations consisted of the following sequences: Sagittal STIR, repetition time = 5000 ms, echo time = 29 ms, inversion time = 150, field of view = 20 cm, slice thickness = 4.0 mm, interslice gap = 0.4 mm, matrix = 266 × 512 pixels, one excitation and acquisition time 5.26 min; sagittal and axial T1-weighted sequences; and sagittal and axial fat saturated T1 post contrast sequences. All sagittal images were obtained perpendicular to the line connecting the dorsal aspect of the medial and lateral femoral condyle (Figure 1). Only the sagittal STIR images were analyzed in the present study.

### Image analysis

**Figure 2:**
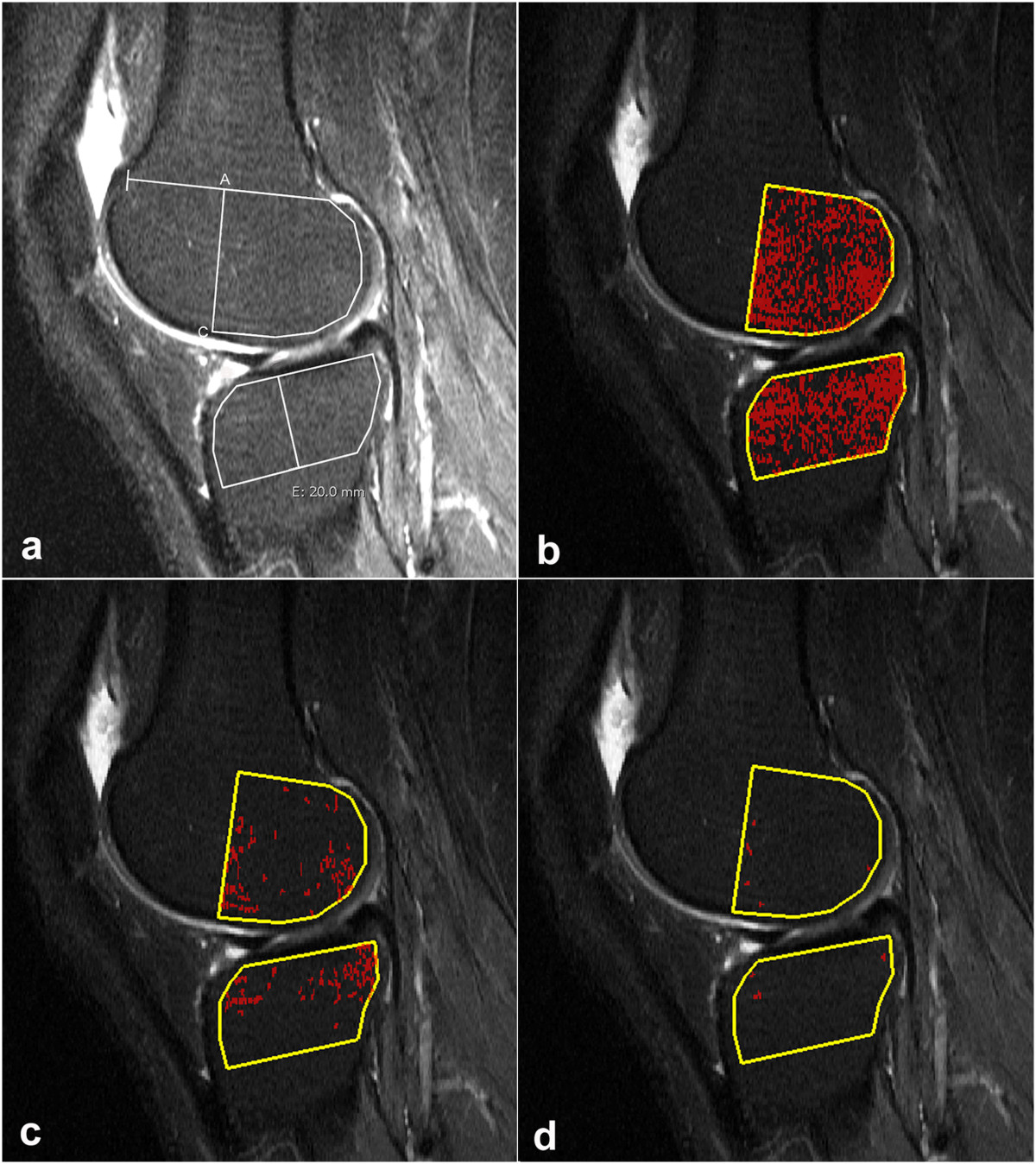
**Sagittal STIR image through the central portion of the lateral femoral and tibial condyle.** **(a)** Illustrates the drawing of reference areas for both manual and computer assisted segmentation (CAS). The dorsal portion of the femoral condyle is outlined by the midpoint (A) between the ventral and dorsal aspects of the physis, the ventral aspect of the meniscus (C) and a line following the articular surface in a distance of 3 mm from the cartilage. The tibial condyle area is outlined by a line following the subcondral bone, a straight line 20 mm distal for the lowest point of the subchondral line and lines ventrally and dorsally 3 mm below the cortical surfaces. The area of the dorsal femoral bone was 815 mm^2^ and the mean signal intensity (SI) 185; standard deviation (SD) 34. The tibial area was 662 mm^2^ with a mean SI of of 186 and SD = 36. The corresponding CAS segmentation is shown in b-d. **(b)** Image obtained corresponding to the mean SI of 187 (SD = 35) in the femur and 187 (SD = 33) in the tibial condyle show an even distribution of marked and non-marked pixels. **(c)** Illustrates the use of a threshold corresponding to the mean SI plus one SD; 8% of the pixels have SIs above the threshold. **(d)** Image obtained with a threshold corresponding to the mean SI + 2SD; less than 1% of the pixels have SIs above the threshold.

**Figure 3:**
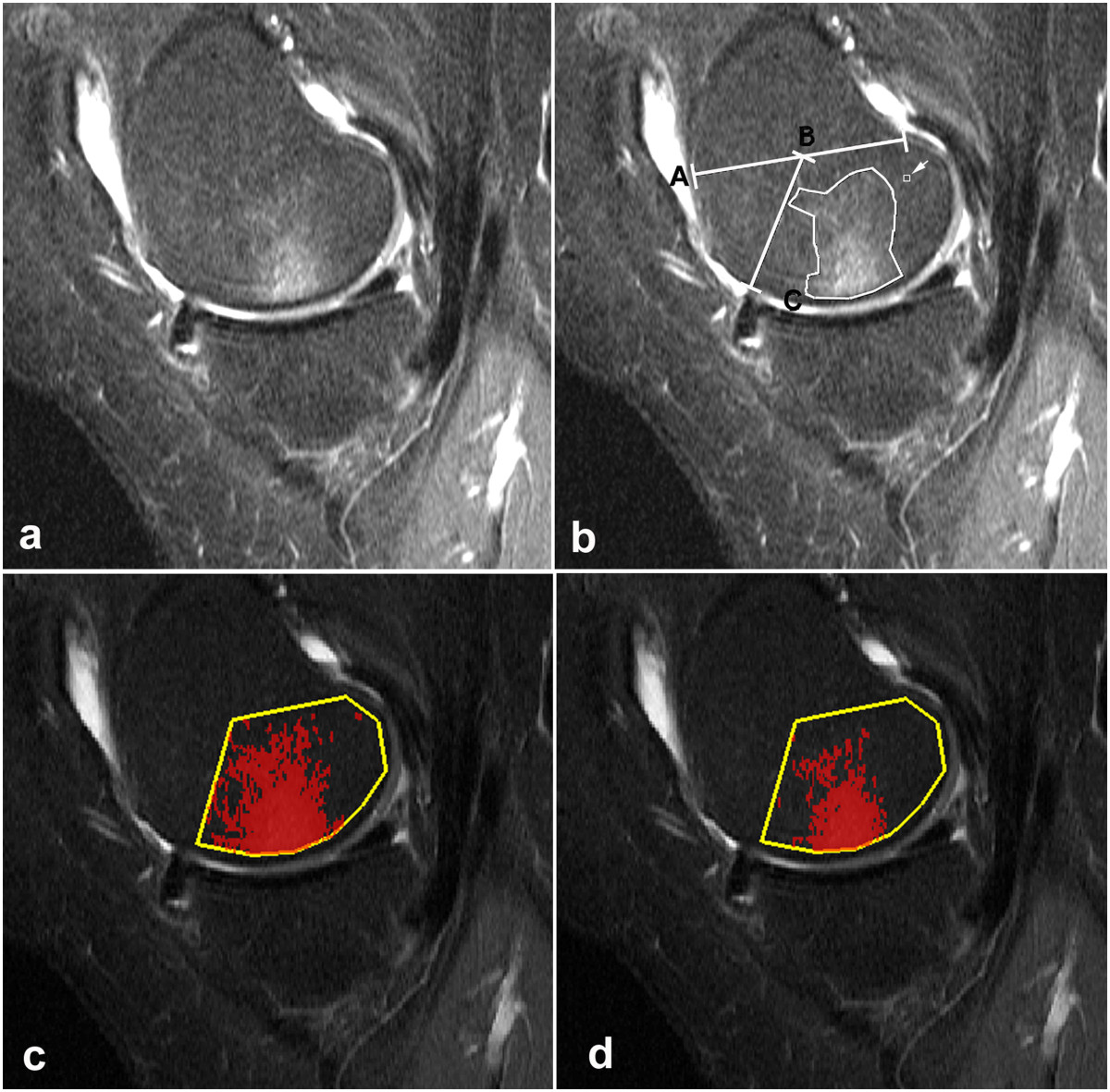
**Sagittal STIR image medially in the same 61-year-old female as shown in Figure** 2 **.** **(a, b)** The posterior femoral region is delineated via points A-C. There are ill-defined increased SI areas in the bone marrow (bone marrow lesion (BML)) of the medial femoral condyle as part of medial osteoarthritis (OA). **(b)** The manually segmented area of BML with SIs above the threshold value (219) was 334 mm^2^ being finally adjusted by measuring the SIs along the segmentation borders using a small rectangular area (arrow). (c, d) The computer assisted segmentation (CAS) of the BMLs is shown with a threshold corresponding to the mean SI plus 1 SD **(c)** and 2 SDs **(d)**, respectively. The shape and extend of the area of pixels above the threshold displayed in **(d)** corresponds better to the area of manual segmentation **(b)** than the area in **(c)**, 183 mm^2^ and 304 mm^2^, respectively.

MR images were anonymized and assessed independently by an experienced musculoskeletal radiologist (NE) and a radiological registrar (FKN). The volume of BMLs was measured by two methods using threshold values of the SI obtained from not affected bone marrow laterally (Figure 2) to assess SI of BML medially; one with manual segmentation (MS) and one with computer assisted automatic segmentation (CAS).The individual assessments were preceded by an initial training and calibration session of 15 examinations not included in the reliability readings, encompassing 10 knees with varying size of BML and five without BML. Using both methods the 15 examinations were assessed in consensus by all authors. The most appropriate reference threshold SI values were determined by testing the reference SI plus one and two SDs in relation to the authors' subjective evaluation of SIs conforming to the definition of BML (Figure 3). By MS, boundary demarcations obtained with the reference SI plus one SD were deemed most representative for the subjective extension of the ill-defined BMLs; by CAS the areas of attenuating voxels had to be obtained with the reference SI plus two SDs to be comparable with visible BML (Figure 3).

**Figure 4:**
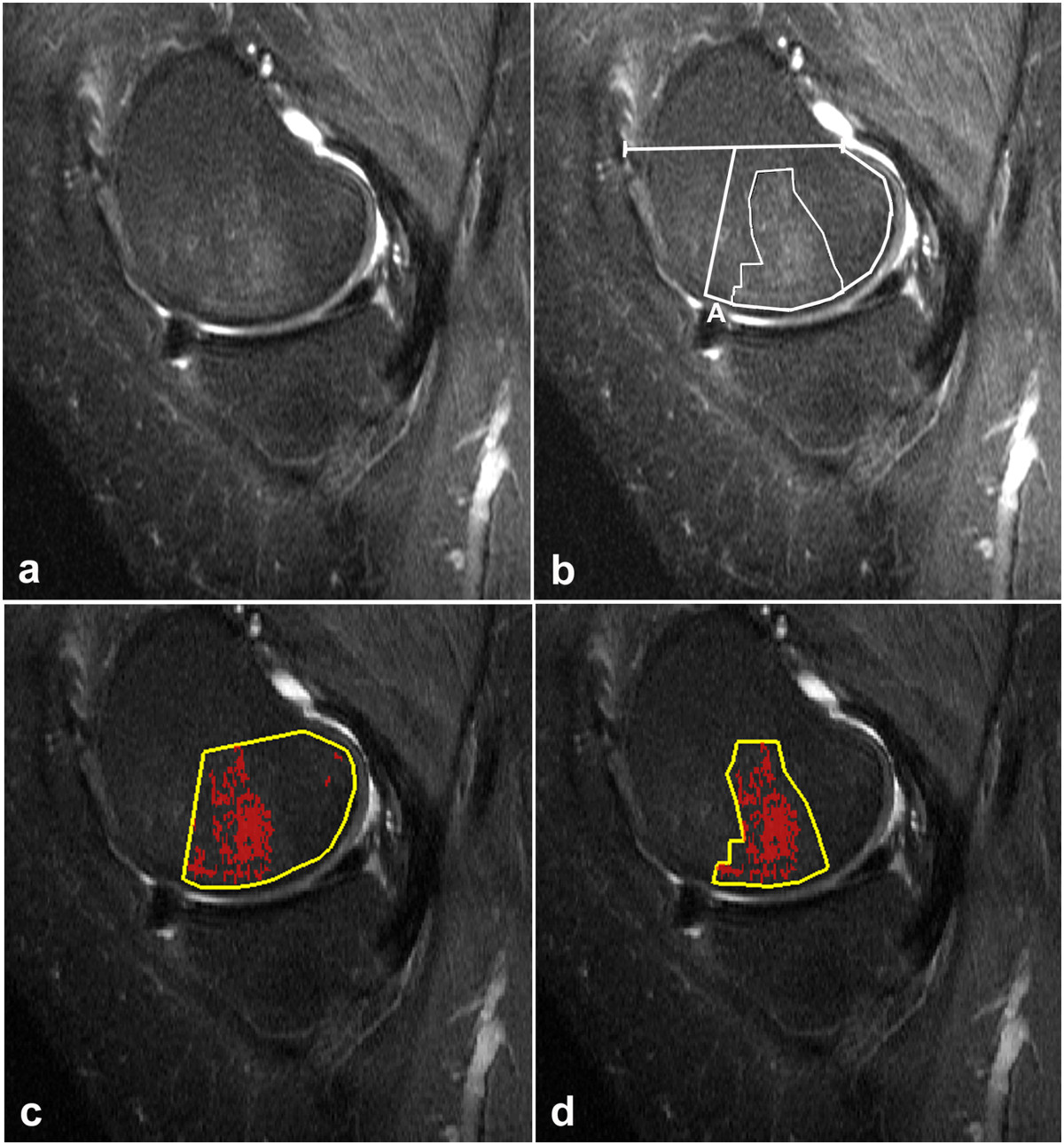
**Comparison of femoral BMLs by the manual and the computer assisted method, respectively. (a)** Sagittal STIR image with ill-defined BMLs of the medial femoral condyle in a female with medial OA. **(b)** The area of BML segmented manually was 357 mm^2^ using a threshold SI of 231 (mean 196 + 35 (1 SD)). The total area of the posterior region of the femoral condyle was 896 mm^2^ and the percentage of BML thus 40% corresponding to BLOKS grade 3 in this single section. **(c)** Computer assisted segmentation of the same BMLs using a threshold SI of 259 (mean 193 + 66 (2 SDs)). The area of pixels above the threshold SI is 123 mm^2^ corresponding to 14% of the total region analysed in this section. **(d)** The same shape and area (357 mm^2^) as used for manual segmentation in **(b)** is drawn; the percentage of pixels above the threshold SI within this area is 32%. (Using a threshold of 226 (mean plus 1 SD) the percentage of pixels above the threshold would increase to 60%; not shown).

**Figure 5:**
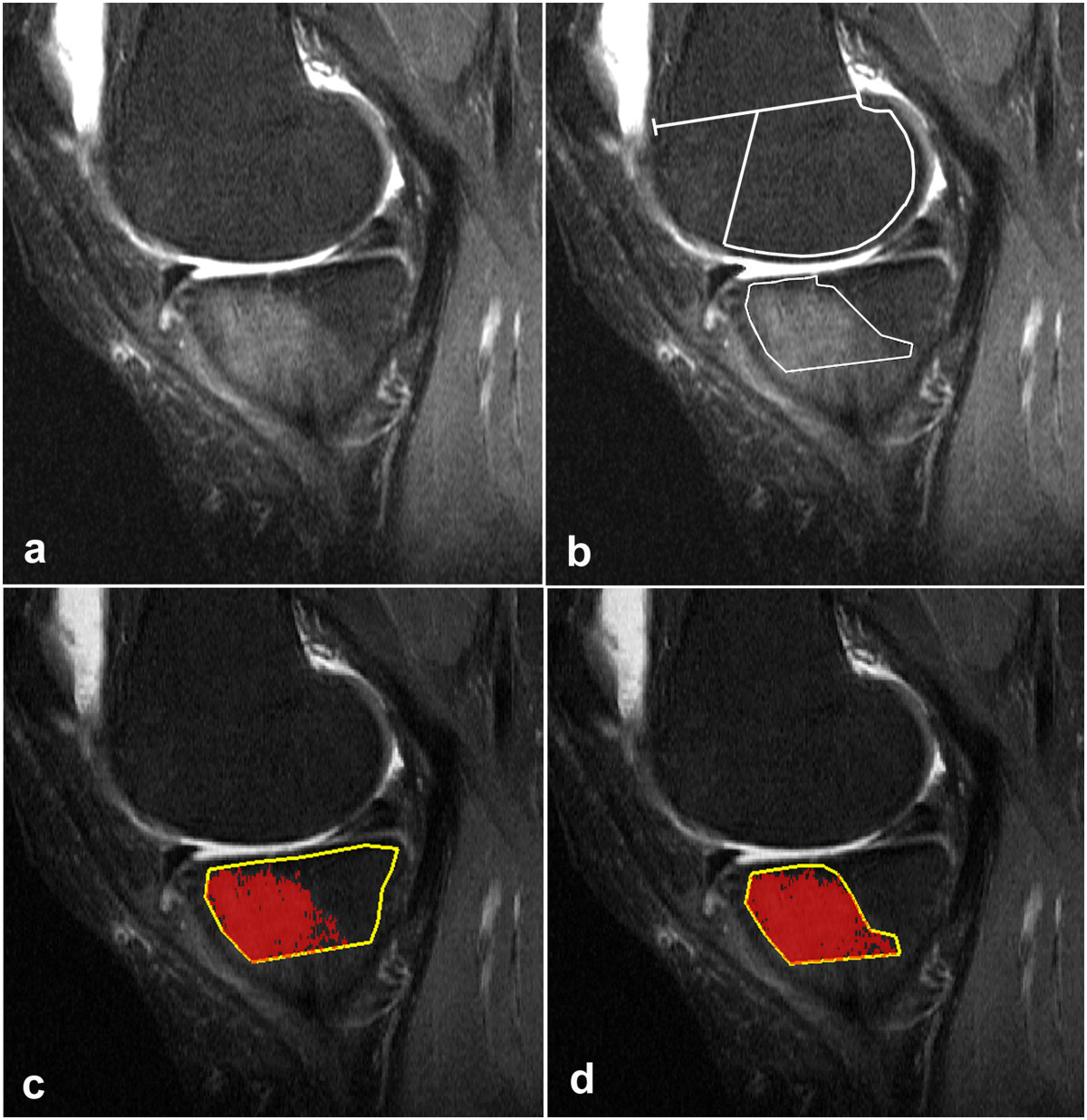
**Comparison of tibial BMLs by the manual and the computer assisted method, respectively. (a)** Sagittal STIR sequence with BMLs of the medial tibial condyle in a female with medial OA. **(b)** Manual segmentation using a threshold SI of 225 (mean 172 + 53 (1 SD)) resulted in a BML area of 442 mm^2^. The total tibial condyle area was 686 mm^2^ and the percentage of BML thus 64% corresponding to BLOKS grade 3 [20] in this single section. **(c)** Computer assisted segmentation of the same BMLs using a threshold SI of 260 (mean 168 + 88 (2 SDs)). The area of pixels above the threshold SI was 316 mm^2^ corresponding to 48% of the condyle area. **(d)** The same shape and area (442 mm^2^) as used for manual segmentation in **(b)** is drawn; the percentage of pixels above the threshold SI within this area is 87%. (Using a threshold of 226 (mean plus 1 SD) the percentage of pixels above the threshold would increase to 93%).

The measurements by both methods were confined to four articular subregions of the knee, the posterior 2/3 of the medial and lateral femoral condyles delineated on sagittal images according to Hunter et al. 2008 [20] as well as the medial and lateral tibial plateaus (Figures 2, 3, 4 and 5). The distal demarcation of the subregions of the tibial plateaus was corresponding to a straight line 20 mm distal to the lowest point of the subchondral bone (Figures 2 and 5). The medial and lateral femoral condyle subregions were delineated excluding sections with partial volume SI derived from the soft tissues in the intercondylar area and at the outer margins of the condyles (Figure 1). Similarly, the peripheral slices of the medial and lateral tibial plateau subregions with partial volume SIs from soft tissue and sections containing portions of the tibial eminence were excluded. With a slice thickness of 4 mm and interslice gap of 0.4 mm, only 3-4 slices of the medial femoral condyle remained for analysis due to obliquity of the medial contour of the medial femoral condyle (Figure 1). Thus, three slices were used medially and the central one or two slices were used laterally.

MS was performed on a standard radiological workstation (Agfa Impax, Belgium, version 6.3.1.8000) with 2 K Bracco screens using standard graphic tools. The CAS evaluations were performed on a PC with high-resolution screens in a MATLAB (the Works, Sweden) graphic user interface developed by one of the authors (DP). Evaluation by MS and CAS, respectively, was performed independently with a time interval of no less than four weeks between the MS and CAS. All examinations were re-analyzed by FKN using both methods at least three months after the first evaluations.The first step in both methods was to delineate the boundaries of the subregions manually by marking multiple points along the articular surfaces in each of the relevant slices to obtain an average reference SI value with SDs laterally and a BML volume estimate medially (Figures 2, 3, 4 and 5). By MS the reference SI values and SDs were obtained from one central lateral slice whereas two adjacent central slices were used by CAS with digital automatically calculated average reference SIs and SDs.

In the third step of MS the boundaries of the BMLs in each of the three central slices of the medial femoral condyles and tibial plateaus were subjectively outlined by marking multiple points along the ill-defined BML borders. Using a rectangular region of interest (ROI) of 5 mm^2^ the boundaries of the marked BMLs were scanned for areas with SIs above and below the threshold SI and the demarcation points and connected lines were adjusted accordingly (Figure 3). In each slice, the marked areas of BML were recorded and volumes calculated by multiplying with slice thickness including interslice gap. Subsequently, the total volume of BMLs in all three slices was calculated.

In the third step of the CAS method voxels with SIs above the threshold values were calculated digitally and visualized on the images of each slice of the medial subregions. The total three dimensional volumes of BMLs in the selected slices were automatically calculated. Digital calculations of volumes excluded scattered clusters of less than five interconnected voxels regarded as noise.

The total time consumption for each method including threshold estimation, BML-segmentation and processing of results was 30-45 minutes for MS and 15 minutes for CAS, respectively.

The volumes of BML were related to the condyle/plateau volumes of the three slices analysed as percentage BML which were used to determine sensitivity to changes. These relative values obtained by MS were compared with a semi-quantitative BLOKS (Boston Leeds Osteoarthritis Knee Score) grading of the BMLs [20]. The percentage of BML volume in the three slices at baseline and follow-up MRI were according to BLOKS divided in four groups: none, <10%, 10-25% and >25%. The number of joints with definite changes over time by the two segmentation methods and BLOKS grading, respectively, were compared.

### Statistical analysis

Data were analyzed using STATA (version 12, Stata Corp., College Station, X, USA) and Analyze-IT software (Analyse-it for Microsoft Excel (version 2.20) Ltd.; 2009). The reference SI values obtained by MS and CAS, respectively, were compared using Student’s t-test. The inter- and intra-observer reliability was assessed by Bland-Altman analyses, using plots, bias and 95% confidence intervals (CIs). Agreement was measured by comparing the absolute as well as the relative BML volume (percentage of the entire condyle volume) in all of the 44 femoral and tibial condyles assessed irrespective of baseline or follow-up status. The BML volumes obtained by MS and CAS, respectively, were compared using Bland-Altman analysis and Spearman’s correlation test.

The inter- and intra-observer variations regarding the threshold values measured laterally were also compared using Bland-Altman plots, bias and 95% CIs as they impacted on the calculations of BML volumes.

Sensitivity to change was estimated by comparing the relative BML volumes at baseline and follow-up for MS and CAS, respectively, based on the evaluations of both assessors. Any change in relative BML volume at follow-up exceeding the 95% limits of agreement for baseline BMLs was considered significant (minimal detectable change = MDC) in accordance with previous studies [27].

## Results

The mean SI of the normal bone marrow obtained by CAS and MS were almost identical being 182.1 and 181.6, respectively, with a mean difference of 0.5 (95 % CI -9.6 to 10.6, p = 0.92). The inter- and intra-observer agreements for reference threshold values were high with small bias values (Table 1).

**Table 1:** **Inter- and intra-observer agreement for reference threshold values, and absolute and relative BML volume in 44 knees**

Observer agreements											
Area	Method	Observer agreement	Reference values			Absolute BML			Relative BML		
			Bias	95% CI		Bias, mm^3^	95% CI		Bias, %	95% CI	
Femur	CAS	Inter-observer	1.7	-0.2	3.6	15	-60	89	0.01	-0.41	0.43
		Intra-observer	-0.1	-1	0.8	67	13	121	-0.10	-0.42	0.21
	Manual	Inter-observer	-0.1	-4.6	4.5	7	-129	144	-0.36	-1.45	0.73
		Intra-observer	-1.8	-3.6	-0.1	142	-41	325	1.15	-0.46	2.76
Tibia	CAS	Inter-observer	-0.1	-4.6	4.5	-74	-140	9	-0.10	-0.70	0.51
		Intra-observer	-1.8	-3.6	-0.1	61	28	94	0.22	-0.04	0.49
	Manual	Inter-observer	1.7	-0.2	3.6	-83	-209	44	-0.26	-0.82	1.35
		Intra-observer	-0.1	-1	0.8	129	6	251	1.18	-0.06	2.41

CAS = computer assisted segmentation.

Data obtained by Bland-Altman tests, bias and 95% confidence interval (CI).

### BML volume assessment

Based on MS 13 femoral condyles and 15 tibial plateaus did not show BMLs. In total 13 of the 44 knees were without BML. In the 28 condyles/plateaus without BML by MS the CAS method detected clusters of more than four pixels exceeding the threshold in all examinations (Figure 2). For all examinations, the BML volumes obtained by CAS were lower than those obtained by MS, especially in the tibia (Figure 5). The average median BML volumes in the femur were 1319 mm^3^ and 1828 mm^3^, and in the tibia 941 mm^3^ and 2097 mm^3^ using CAS and MS, respectively (Table 2). This difference was partly caused by inclusion of areas not exceeding the computerized thresholding in the manually segmented areas (Figures 3, 4 and 5). The percentage of BML volume in relation to the condyle volume analysed (relative BML-involvement) was also lower by CAS than by manual segmentation (Table 3).

**Table 2:** **Absolute values of BML volume by the CAS and the manual segmentation (MS) method**

Absolute values of BMLs				
Area	Segmentation method	BML volume, median (25th - 75th quartile), mm^3^		Average BML volume, median (25th - 75th quartile), mm^3^
		Observer 1	Observer 2	
Femur	CAS	1326 (459 - 2743)	1300 (519 - 2971)	1319 (509 - 2857)
	Manual	1756 (777 - 3586)	2165 (715 - 3683)	1828 (725 - 3634)
Tibia	CAS	687 (276 - 2546)	839 (286 - 2518)	941 (273 - 2532)
	Manual	2068 (950 - 3780)	1940 (761 - 4453)	2097 (851 - 4116)

CAS = computer assisted segmentation.

In 32/31 femoral condyles and 31/29 tibial plateaus (CAS/MS respectively).

**Table 3:** **Relative values of BML volume by the CAS and the MS method**

Relative BML volumes			
Area	Segmentation method	% BML involvement, median (25th - 75th quartile)	
		Observer 1	Observer 2
Femur	CAS	10.5 (3.8 - 25.6)	9.5 (3.7 - 26.6)
	Manual	14.9 (5.6 - 31.2)	14.8 (5.0 - 36.9)
Tibia	CAS	6.5 (2.7 - 21.9)	7.7 (2.6 - 19.5)
	Manual	19.7 (7.9 -34.1)	17.5 (7.8 - 35.6)

CAS = computer assisted segmentation.

In 32/31 femoral condyles and 31/29 tibial plateaus (CAS/MS respectively).

The observer variation of absolute BML volumes varied between the methods (Table 1). In the femur, the inter-observer variations were acceptable with a bias of 15 mm^3^ (95% CI -60 to 89) and 7 mm^3^ (95% CI -129 to 144) by CAS and MS, respectively. The variation was larger in the tibia; the inter-observer bias was -74 mm^3^ (95% CI -140 to 9) and -83 mm^3^ (95% CI -209 to 44) by CAS and MS, respectively. The intra-observer variation was inferior to the inter-observer variation (Table 1).

Comparing the relative BML volumes (Table 1), the systematic bias for inter-observer variation was close to zero and the CIs were narrow. Thus, the largest inter-observer variation was -0.36% (95% CI -1.45 to 0.73) (femur, MS), the largest intra-observer variation was 1.18% (95% CI -0.06 to 2.41) (tibia, MS).

Bland-Altman analysis of the BML obtained by CAS and MS, respectively, yielded a systematic bias of -438 mm^3^ (95% CI -702 to -174) and -853 mm^3^ (95% CI -1265 to 440) in the femur and tibia, respectively; for the relative BML involvement, the systematic bias was -3.7% (95% CI -6.1 to -1.3) and -8.4% (95% -12.4 to -4.4) in the femur and tibia, respectively. However, the absolute and relative BML volumes obtained by CAS and MS were significantly correlated, ρ values for BML in the femur being 0.87 and 0.85 for absolute and relative volumes, respectively, and 0.82 and 0.81 in the tibia, respectively (p < 0.0001 for all analyses).

### Sensitivity to change

Using CAS, the relative BML involvement of the analysed condyle volume had to decrease by at least 2.0 – 4.5% or increase by 1.8 – 4.7% from baseline to follow-up to be significant (Table 4). The 95% limits of agreement were wider using MS; the decrease in relative BML involvement had to be at least 6.5 – 6.9% and the increase at least 6.2 - 8.2%. A significant change was observed in 13 of the 22 knees, 11/22 femoral condyles and 8/22 tibial plateaus using CAS, and in 10 knees, 7/22 femoral condyles and 9/22 tibial plateaus, using MS. Progression of OA BML changes was observed in six patients using both CAS and MS whereas seven patients revealed regression by CAS compared to 4 patients using MS. In comparison the BLOKS grading changed in 9 knees, 7/22 femoral condyles and 4/22 tibial plateaus, from baseline to follow-up. Progression was observed in 3 femoral condyles and 2 tibial plateaus, regression in 4 femoral condyles and 2 tibial plateaus.

**Table 4:** **The 95% limits of inter-observer agreement of relative BML volumes at baseline**

95% limits of agreement				
Area	Segmentation method	Inter-/intra-observer agreement	95 % limits of agreement	
Femur	CAS	Inter-observer	-2.0	1.8
	Manual	Inter-observer	-6.5	6.2
Tibia	CAS	Inter-observer	-4.5	4.7
	Manual	Inter-observer	-6.9	8.2

CAS = computer assisted segmentation.

Based on the 22 baseline examinations used to determine sensitivity to change over time.

## Discussion

This study tested and analysed two quantitative methods for measuring KOA related BMLs, one based on CAS and one based on MS. Both methods included threshold values defined by the SI in healthy bone marrow and the threshold values obtained by the CAS and MS method were almost similar indicating that the underlying basis for the measurements was comparable. There was a high degree of inter-observer agreement, confirmed by small bias values and narrow 95% CIs using Bland-Altman analyses. The intra-observer agreement was acceptable, but inferior to the inter-observer agreement, especially by MS. This could be explained by a long period (>3 months) between the two measurements and by the first but not the second image analysis being preceded by a calibration session. The CAS results differed less in accordance with a more user-independent method.

The high inter-observer agreement resulted in small MDC values, especially by the CAS method. Progression of BMLs exceeding 1.8 – 4.7% or regression below -2.0 - -4.5% of the baseline BML volume were significant using CAS. This occurred in 13 of the 22 patients (59%) (11 femoral condyles and 8 tibial plateaus). In comparison, significant changes were observed in 10 of the 22 patients (49%) (7 femoral condyles and 9 tibial plateaus) by MS. Both methods thus seem usable for detecting even small changes occurring during a relatively short follow-up (median 11.1 months). There were, however, important differences between the two segmentation methods. The CAS method measured the exact volume of pixels above the defined threshold whereas bone marrow with visually altered signal characteristics was measured by MS. The individual BML often consists of a variety of histological pathologies, e.g. necrosis, fibrosis, haemorrhage, abnormal trabeculae and microfractures in various stages of healing. Hence, signal intensities within a BML are often heterogeneous with areas above and areas below the defined threshold which cannot be separated manually. Measurement of the exact pixel volume above a defined threshold by the CAS method might therefore be the most precise and furthermore least time-consuming tool.

An MS method similar to ours had been used in a study of patello-femoral OA [27] but without the use of threshold values based on normal bone marrow. The natural fluctuation of BMLs over 6 – 12 weeks was analysed and sensitivity to change calculated using Bland-Altman 95% limits of agreement as MDC values. These limits were wide as all measurements were reported in absolute values as mm^3^. Despite this, 41% of the knees showed changes greater than the MDC using axial slices. It is possible that a use of relative BML involvement and/or threshold values, as in the current study, might have resulted in a higher number of significant changes. However, their results based on sagittal slices were inferior to those obtained by axial slice orientation, confirming that this slice orientation is not optimal for evaluating the patello-femoral joint [27].

The CAS method has been tested previously. Mayerhoefer et al. [24] analysed 10 patients with “bone marrow oedema” and reported excellent intra-observer agreement. However, the method was time-consuming and has to our knowledge subsequently only been used in a study comparing bone marrow oedema and contrast enhancement, concluding that STIR is the optimal method for determining BML size [34].

Two other studies have used CAS methods to detect and quantify BML [25, 26]. The method described by Pang et al. [25] included a fully automatic segmentation of BMLs where a predefined false discovery rate was used to determine the threshold SI. The results were promising, including a correlation between the measured BML volumes by CAS and BLOKS grading corresponding to the tibial plateau. However, their observer agreement was low in the tibial plateaus. A comparison of femoral BMLs was not possible due to different volumes of interest in the CAS method and by BLOKS grading. The method by Ratzlaff et al. [26] was as our CAS method based on threshold SI values obtained from normal bone. Their CAS volume results were significantly correlated with a pre-determined WORMS (Whole-Organ Magnetic Resonance Imaging Score) grading. The sensitivity to detect change of BML over time has not been evaluated using these two methods although the method described by Pang et al. [25] has been used in two longitudinal studies [13, 35].

Previously, quantification of BML has mostly been performed using semi-quantitative methods such as the BLOKS, WORMS and KOSS (Knee Osteoarthritis Scoring System) grading [3, 18, 20]. These methods have been shown to be able to detect BML changes within two years [36, 37]. In the present study significant changes occurred in 13 knees by CAS whereas only 9 knees changed BLOKS grading. This may indicate that the CAS method is more sensitive to detect change than the BLOKS grading. A comparison of our detected BML volumes with other semi-quantitative grading systems was not possible as their division of subchondral regions was different from the one used in the present study. However, WORMS and BLOKS grading have been shown to be relatively comparable with a high reliability for most BML scores [36]. In cross-sectional comparisons of BMLs the two methods gave similar results regarding prevalence and severity of BMLs. However, in longitudinal studies BML scoring according to WORMS was found superior as it was the easiest to perform and gave a better prediction of later cartilage loss [36].

Strengths of the present study include the blinded readings and the high inter-observer agreement resulting in small MDC values, especially by CAS.

The study has several limitations: The STIR images evaluated were obtained using a 1.5 T scanner. We did not evaluate other magnetic field strengths or sequences, where the performance may be different. The patello-femoral joint – a common site for BML in OA - was not examined due to the sagittal slice orientation. Although we compared our results with BLOKS grading, comparison with other semi-quantitative grading systems such as WORMS and KOSS grading was not performed.

The different volumes of BML obtained by CAS and MS, respectively, may partly be due to the different threshold values based on the mean SI value of normal bone plus one SD by MS and two SD by CAS. Although these threshold values were carefully selected by a consensus reading of all authors, it is possible that the use of the mean SI + 1.5 SD by the CAS method would have resulted in more comparable volumes/values; this was not tested. However, MS will always include voxels below the threshold SIs.

Perspectives: OA is increasingly considered an inflammatory disorder and DMOADs are emerging [28, 29]. In the development and testing of new DMOADs it is essential that imaging methods are sensitive to detect minor changes over time. This will not only enable shorter follow-up but also reduce the number of patients needed to obtain significant results. The current CAS method can probably be of considerable value in therapeutic studies.

## Conclusions

CAS was a reliable method for measuring BML and more sensitive to detect changes over time than MS. The BML volumes measured by the two methods differed but were significantly correlated.

## Authors’ information

FKN: Department of Radiology, Aarhus University Hospital, Noerrebrogade 44, 8000, Aarhus, Denmark: Phd student within MRI of knee disorders including osteoarthritis.

NE: Musculoskeletal radiologist, DMSc and Professor. Department of Radiology, Aarhus University Hospital, Noerrebrogade 44, 8000, Aarhus, Denmark.

DP: Phd, Department of Biomedical Engineering, Aarhus University Hospital, Noerrebrogade 44, 8000, Aarhus, Denmark.

AGJ: Musculoskeletal radiologist, DMSc and Professor. Department of Radiology, Aarhus University Hospital, Noerrebrogade 44, 8000, Aarhus, Denmark.

## Authors’ original submitted files for images

Below are the links to the authors’ original submitted files for images. Authors’ original file for figure 1 Authors’ original file for figure 2 Authors’ original file for figure 3 Authors’ original file for figure 4 Authors’ original file for figure 5

## Abbreviations

BML: Bone marrow lesion
CAS: Computer assisted segmentation
MS: Manual segmentation
KOA: Knee osteoarthritis
SD: Standard deviation
SI: Signal intensity.
