# Supplementary figures and images for: Measurement of bone marrow lesions by MR imaging in knee osteoarthritis using quantitative segmentation methods – a reliability and sensitivity to change analysis
Source: BMC Musculoskelet Disord. 2014 Dec 20;15:447. doi: 10.1186/1471-2474-15-447 (PMC4364568; doi:10.1186/1471-2474-15-447)

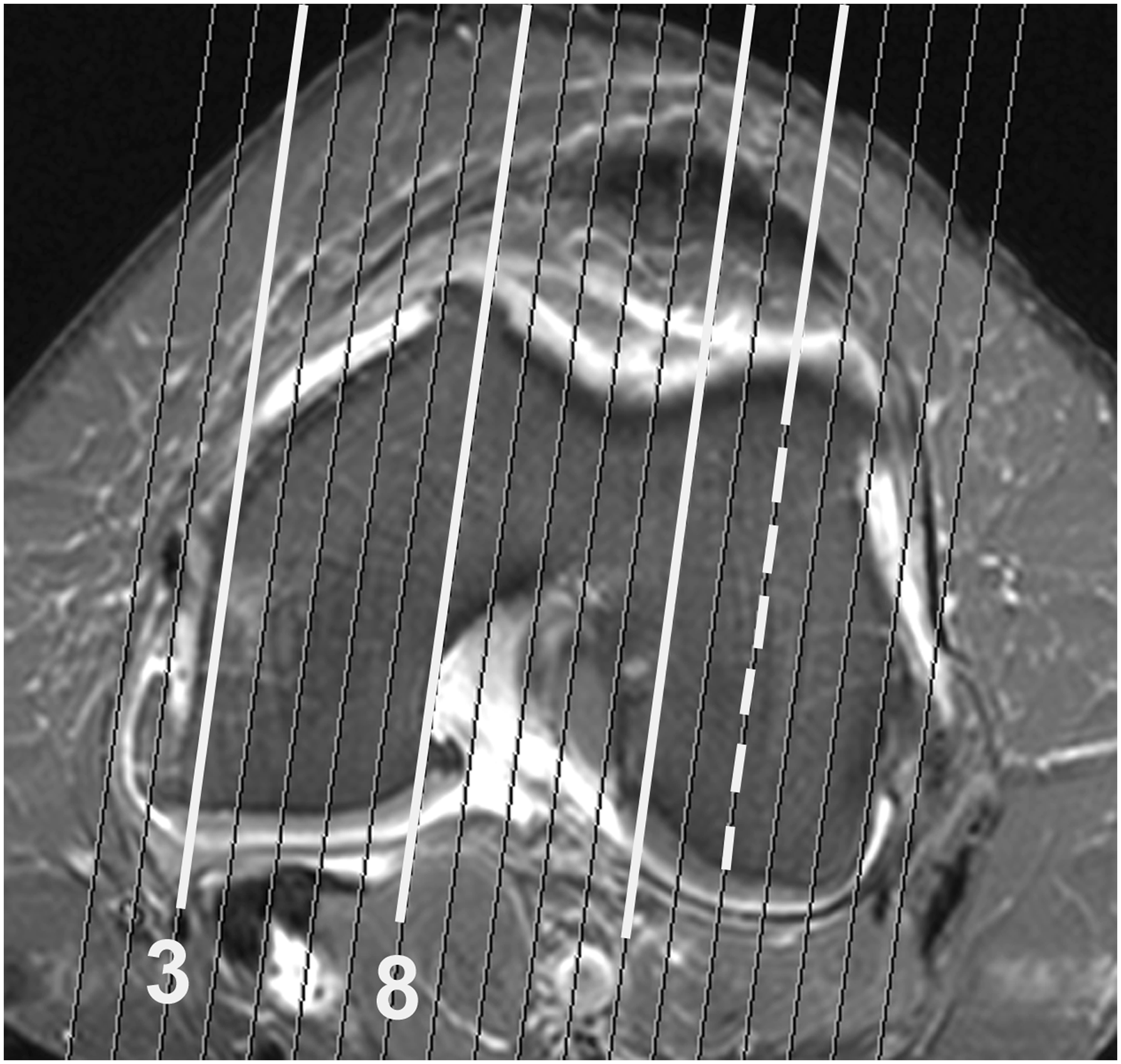

Supplement: Supplementary file 1 — Authors’ original file for figure 1 [file 12891_2014_2386_MOESM1_ESM.tif]

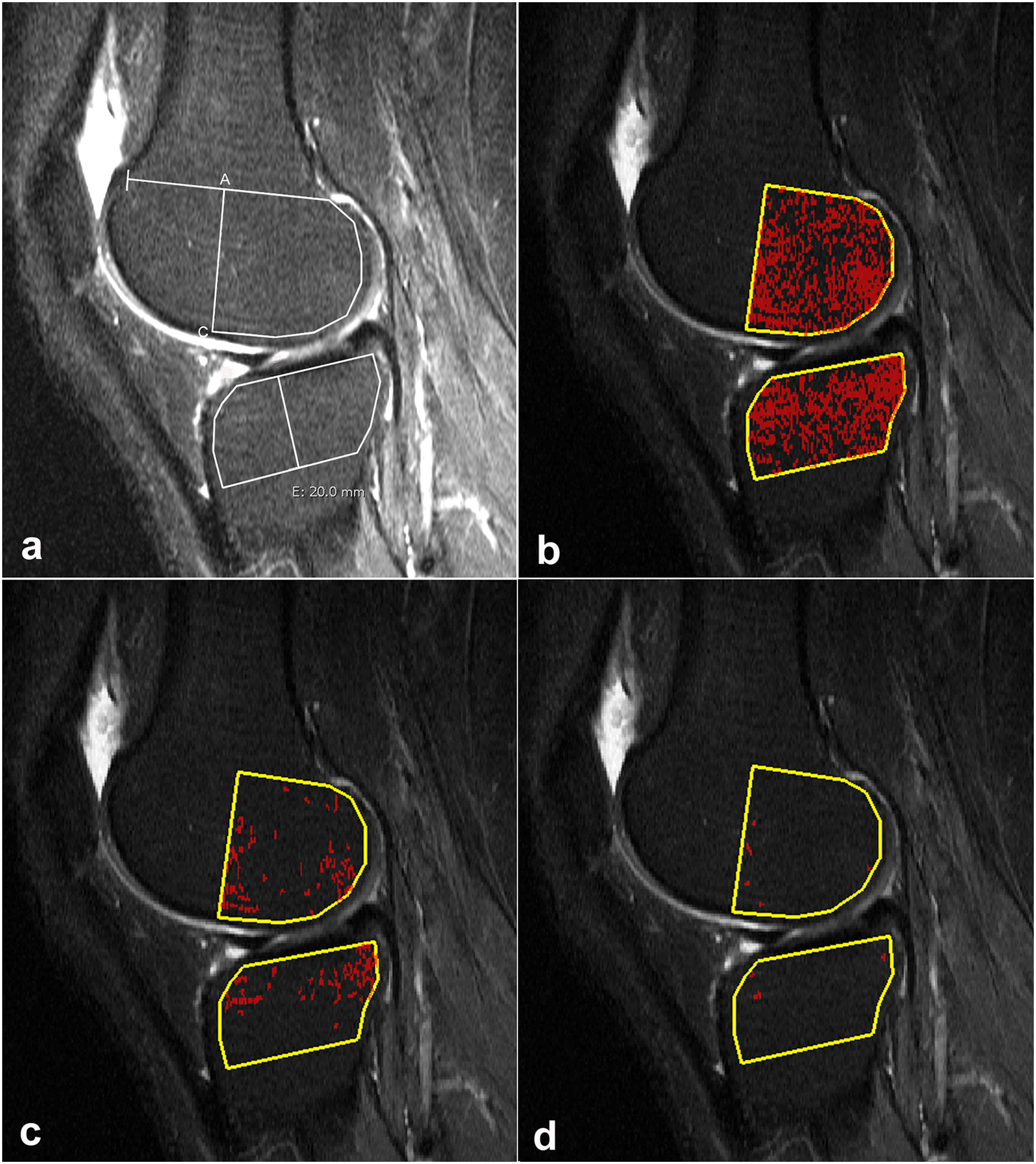

Supplement: Supplementary file 2 — Authors’ original file for figure 2 [file 12891_2014_2386_MOESM2_ESM.tif]

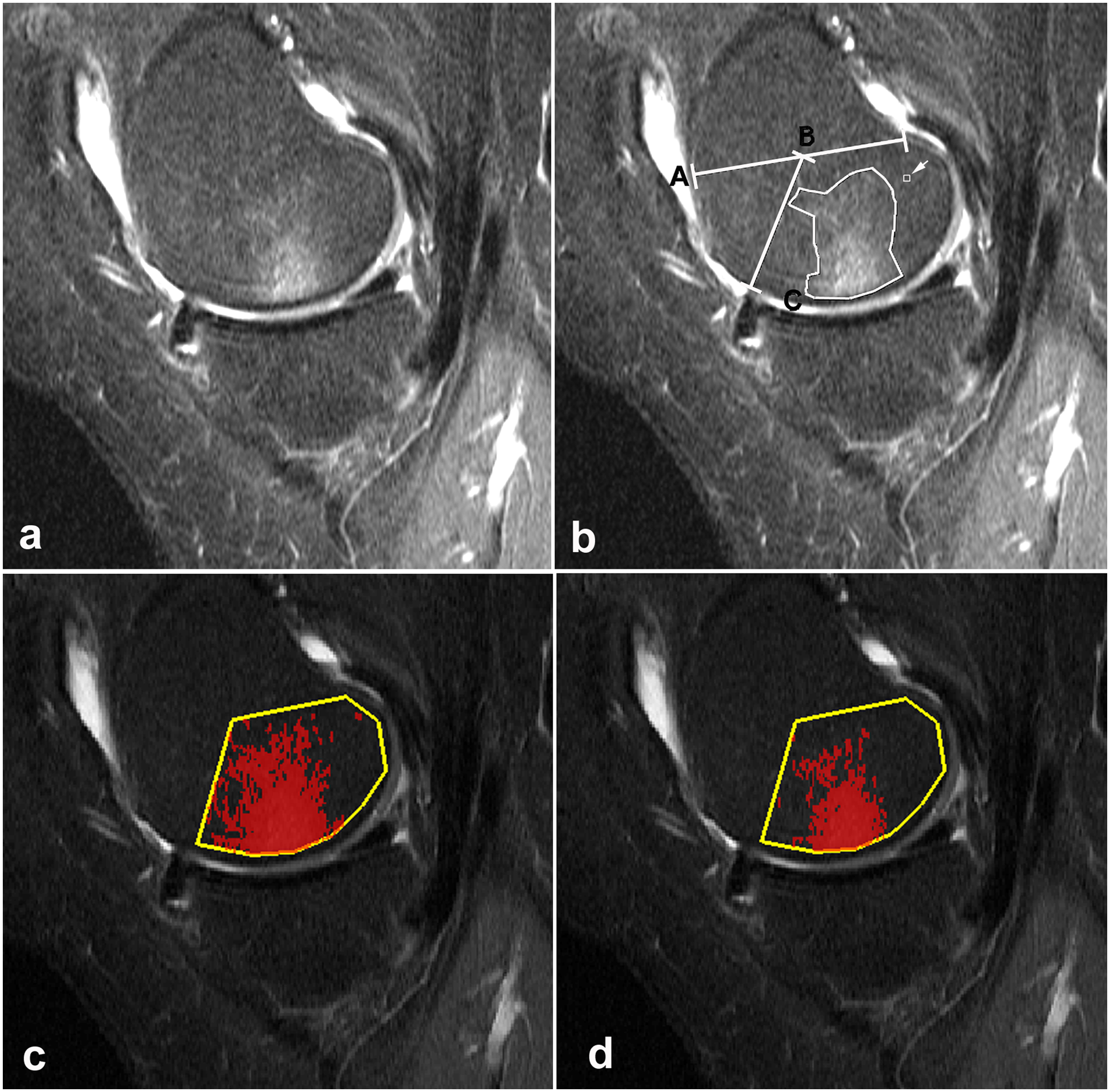

Supplement: Supplementary file 3 — Authors’ original file for figure 3 [file 12891_2014_2386_MOESM3_ESM.tif]

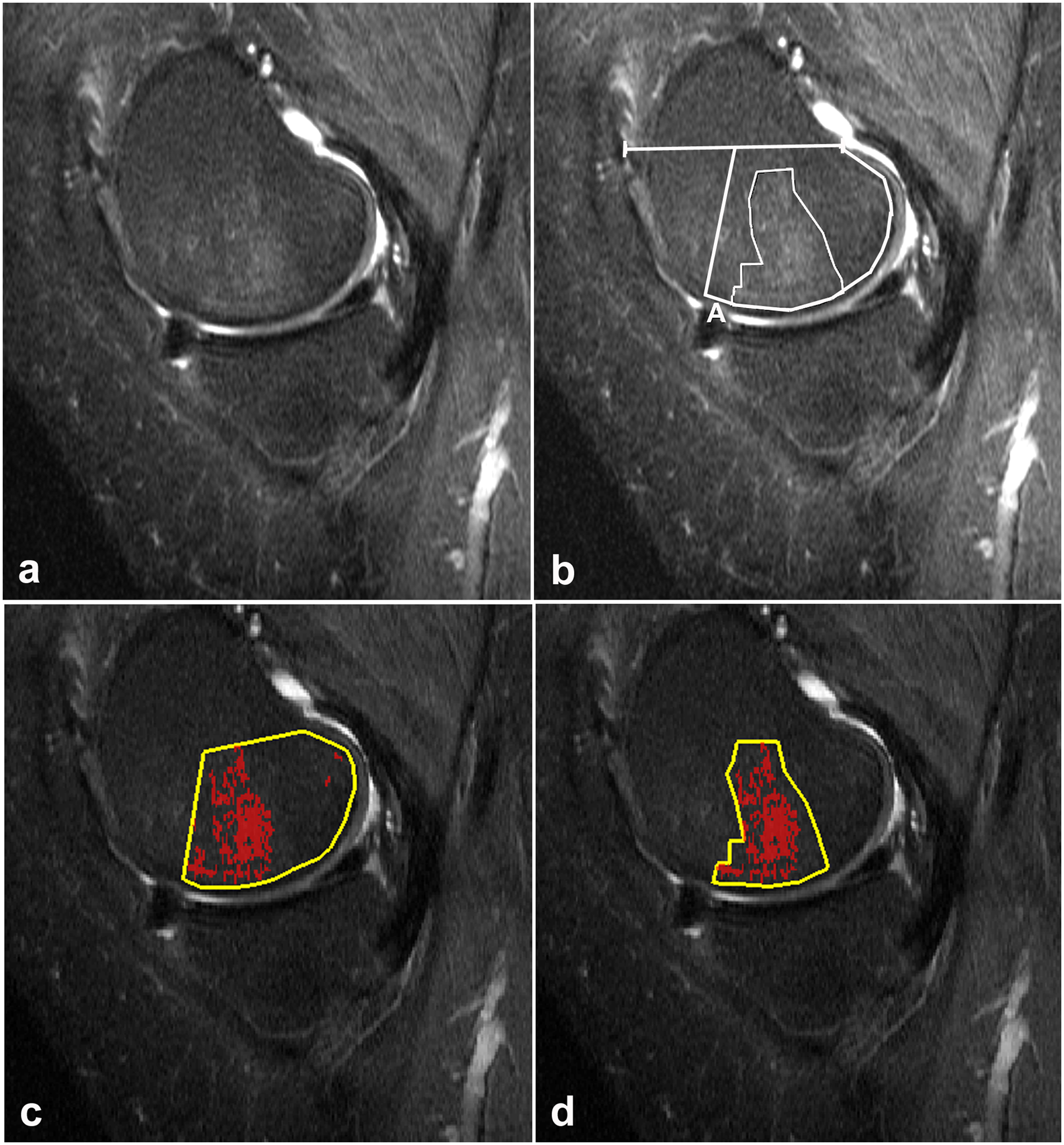

Supplement: Supplementary file 4 — Authors’ original file for figure 4 [file 12891_2014_2386_MOESM4_ESM.tif]

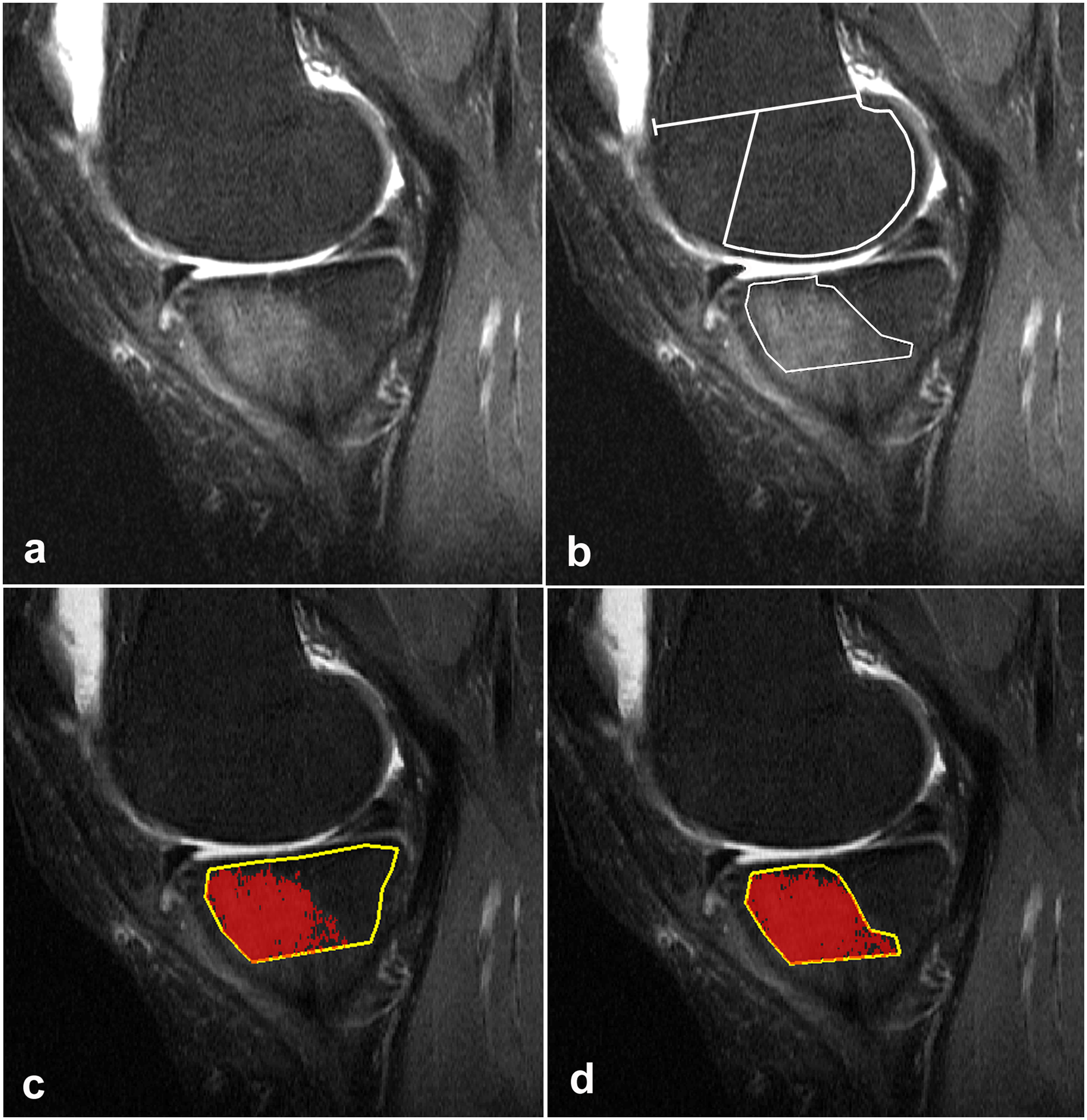

Supplement: Supplementary file 5 — Authors’ original file for figure 5 [file 12891_2014_2386_MOESM5_ESM.tif]
